# Practice makes imperfect: stronger implicit interference with practice in individuals at high risk of developing Alzheimer’s disease

**DOI:** 10.1007/s11357-023-00953-9

**Published:** 2023-10-10

**Authors:** Shao-Min Hung, Sara W. Adams, Cathleen Molloy, Daw-An Wu, Shinsuke Shimojo, Xianghong Arakaki

**Affiliations:** 1 Waseda Institute for Advanced Study, Waseda University, Tokyo, Japan; 2 Faculty of Science and Engineering, Waseda University, Tokyo, Japan; 3 Biology and Biological Engineering, California Institute of Technology, Pasadena, CA USA; 4 Cognition and Brain Integration Laboratory, Department of Neurosciences, Huntington Medical Research Institutes, Pasadena, CA USA; 5 Computation and Neural Systems, California Institute of Technology, Pasadena, CA USA

**Keywords:** Pre-symptomatic Alzheimer’s disease, Implicit processing, Practice, Aging

## Abstract

Early screening to determine patient risk of developing Alzheimer’s will allow better interventions and planning but necessitates accessible methods such as behavioral biomarkers. Previously, we showed that cognitively healthy older individuals whose cerebrospinal fluid amyloid/tau ratio indicates high risk of cognitive decline experienced implicit interference during a high-effort task, signaling early changes in attention. To further investigate attention’s effect on implicit interference, we analyzed two experiments completed sequentially by the same high- and low-risk individuals. We hypothesized that if attention modulates interference, practice would affect the influence of implicit distractors. Indeed, while both groups experienced a strong practice effect, the association between practice and interference effects diverged between groups: stronger practice effects correlated with more implicit interference in high-risk participants, but less interference in low-risk individuals. Furthermore, low-risk individuals showed a positive correlation between implicit interference and EEG low-range alpha event-related desynchronization when switching from high- to low-load tasks. This suggests that lower attention on the task was correlated with stronger interference, a typical phenomenon in the younger population. These results demonstrate how attention impacts implicit interference and highlight early differences in perception between high- and low-risk individuals.

**Supplementary Information:**

The online version contains supplementary material available at 10.1007/s11357-023-00953-9.

## Introduction

Because current options for addressing Alzheimer’s disease are limited to preventative measures or palliative treatments, diagnostic methods that can screen for individuals at high risk of cognitive decline are critical for minimizing disease progression and allowing patients to make decisions about their future care while they are still cognitively healthy. Current methods of identifying such individuals are primarily limited to examining latent pathological changes such as levels of cerebrospinal fluid (CSF) biomarkers tau and amyloid beta (A*β*_42_). Although these biomarkers are well-established in the literature and may precede the onset of symptoms by over a decade [1], their potential use for widespread screening is limited by the expense and invasiveness of the lumbar puncture required for measurement. Behavioral biomarkers could bypass this problem but introduce a new issue: given that most high-risk individuals are cognitively healthy prior to the onset of symptoms, it is extremely difficult to detect disease-dependent cognitive decline at the preclinical stage.

In a recent study, we investigated a possible solution by measuring how cognitively healthy high-risk and low-risk (defined by CSF A*β*_42_/total tau protein ratio) older individuals responded to implicit distracting visual stimuli [2]. Motivated by studies that demonstrate immense interaction between attention and implicit processing in healthy young individuals [3, 4], we hypothesized that early attentional changes could be revealed by evaluating how implicit information was processed in different individuals. Indeed, we found that cognitively healthy older individuals at high risk of cognitive decline experienced interference from an implicit (unseen) stimulus when the task difficulty was high. That is, participants’ reaction time slowed after an incongruent distractor was presented, even when they were not consciously aware of its existence. However, low-risk cognitively healthy older individuals were not susceptible to this implicit distraction. Furthermore, explicit distracting information (e.g., Stroop effect) interfered with the performance of both groups at a similar level. Taken together, these results suggest that early changes in implicit cognition are associated with the risk of cognitive decline, likely linked to Alzheimer’s. Specifically, the inability to suppress implicit distractions may be able to differentiate high-risk individuals from those at low risk of cognitive decline. Furthermore, successful distractor suppression in high-risk individuals depends on how attention is used in a task. Implicit distracting information only interferes with performance when the task load is high, suggesting that the explicit task and implicit sensory information compete for attentional resources. However, a competing theory is that high-risk individuals actively process implicit information. That is, during high-load situations, high-risk individuals direct additional attention to the explicit task, as reflected by a stronger change in alpha frequency in previous studies [5, 6]. Furthermore, such excessive attention spills over to the implicit stimulus, causing active processing.

Since attention deployment is at the core of implicit processing, here we further investigated whether a reduction of task load due to practice interacted with implicit interference. In a related study with healthy young participants, it was shown that short-term practice modulated the strength of implicit interference [4]. These findings provide a solid foundation for the current study, in which we examined existing data from two similar task-switching experiments [2, 5] completed sequentially by the same cognitively healthy older individuals. The participants’ risk status for cognitive decline associated with Alzheimer’s disease had been previously determined for a longitudinal study and defined as cognitively healthy with normal A*β*_42_/total tau protein ratio (CH-NATs, low-risk) or pathological ratio (CH-PATs, high-risk). Our prior EEG study also showed that high-risk and low-risk older individuals exhibited alpha event-related desynchronization (ERD) differences, suggesting different levels of attentional engagement in a working memory task [7], Stroop [6], and task switching [5]. If successful distractor suppression was key to differentiating high-risk and low-risk individuals, we hypothesized that high-risk individuals would especially benefit from having additional attentional resources after practice to suppress distracting implicit information. Similarly, the practice would protect low-risk older individuals from distraction. On the other hand, if high-risk individuals were indeed actively processing implicit information, we hypothesized that the additional attentional resources released from practice would be directed toward implicit cues, which in turn would cause stronger interference.

## Methods

### Participants

We examined data previously collected from thirty-six cognitively healthy older (age range: 53–92) participants with no motor or vision difficulties who completed two prior studies [2, 5] consisting of two separate experimental sessions with identical explicit tasks. In session one, participants performed the behavioral task with concurrent scalp EEG recorded [5]. In session two, participants performed the behavioral task with the addition of an implicit prime [2]. Four individuals who only participated in the second session were excluded from the present study. Each session lasted approximately 50 min. The experimenters and the participants were oblivious to the physiological risk status of the participants (i.e., double-blinded). The institutional review boards (IRB) of the California Institute of Technology (IR19-0963) and the Huntington Medical Research Institutes (HMRI) (Quorum IRB, #27197) approved this study. All participants gave written consent prior to participation. The final analysis included 17 CH-NATs and 19 CH-PATs (see “Physiological status classification” section) for behavioral analysis and 14 CH-NATs and 16 CH-PATs for EEG data analysis (six were excluded because of artifacts). Participants’ demographics, including their age, sex, education year, and neuropsychological test scores, were reported in a previous study [2]. In each analysis, the demographics were comparable between the two groups (Table 1, all *p* > .05).

**Table 1:** Demographics, post-study subjective effort exertion report, resting pulse pressure, and heart rate of CH-NATs (low-risk) and CH-PATs (high-risk)

Behavioral data demographics	CH-NATs (low-risk)	CH-PATs (high-risk)
Total number	17	19
Age	77.29 (8.01)	74.11 (9.22)
Gender	12 females, 5 males	16 females, 3 males
Education year	16.18 (2.10)	16.89 (2.56)
Days between two sessions	15.29 (1.68)	15.84 (2.87)
Resting pulse pressure (systolic-diastolic)	48.38 (14.00)	50.89 (12.72)
Resting heart rate	65.31 (10.56)	66.79 (6.67)
Post-study questionnaire	0: Not at all — 3: Severe	
Did you have any physical troubles during the task?	0.12 (0.33)	0.36 (0.64)
Difficulty: Word responding to word responding	1.12 (0.72)	1.14 (0.74)
Difficulty: Color responding to color responding	1.03 (0.67)	1.22 (0.71)
Difficulty: Word responding to color responding	1.29 (0.79)	1.50 (0.69)
Difficulty: Color responding to word responding	1.32 (0.64)	1.58 (0.65)
How much effort did you use for the task?	1.88 (0.76)	1.97 (0.65)
Do you feel tired after the task?	0.71 (0.85)	0.78 (0.86)

The two groups were comparable in each item with *p* > 0.05. The data is reported as mean (*SD*)

### Physiological status classification

A complete description of classification, including a list of neuropsychological testing, MRI diagnosis of small vessel disease, and the biochemical analysis of lumbar CSF, was detailed in several previous studies [5, 6, 8]. In the structural scans, all participants were awake and none of the participants showed excessive head motion. The inspection for head motion occurred during the scan by visual assessment performed by the MRI technologist and also during the analysis of the images by the radiologist by visual inspection. In short, only participants who were determined to be without (1) cognitive impairment (Clinical Dementia Rating) or (2) psychiatric or neurological disorders were included in the current study. Different cognitive domains including memory, executive function, and language were tested. The CSF samples were run on both Innotest (Innogenetics, discontinued) and MSD platforms (K15121G, MSD) to determine total tau and A*β*_42_. Participants were then classified depending on individual CSF A*β*_42_/total tau ratios compared to a cutoff value (2.7132) derived from a logistic regression model that correctly diagnosed > 85% of clinically probable AD participants [8]. Amyloid/total tau ratios below the cutoff value classified participants as **c**ognitively **h**ealthy–**p**athological **a**myloid/**t**au, i.e., CH-PATs, while participants whose ratios were above the cutoff value were classified as **c**ognitively **h**ealthy–**n**ormal **a**myloid/**t**au, i.e., CH-NATs [2, 5, 6, 8].

### Experimental design

Participants completed both experiment sessions in the same quiet room. The visual stimuli were generated with E-Prime (Psychology Software Tools, Inc.) on a Dell Precision T5610 with a 20′′ screen. The participants’ movements were unconstrained, and they observed the visual stimuli from approximately 50 cm away.

**Fig. 1:**
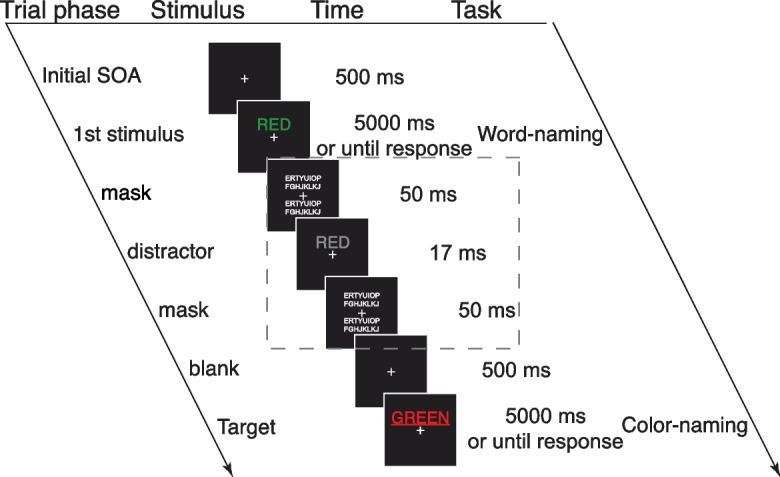
An example trial sequence (modified from [2] Fig. 1B). This is a task-switching trial (from word-naming to color-naming) with a congruent masked distractor. Each trial began with a 500-ms fixation, followed by the appearance of the first word stimulus for 5000 ms or until response. In the second experimental session only, a masked distractor (shown in the dashed box) was presented before the second word stimulus (i.e., target). The masked word was presented for 17 ms either above or below the fixation cross, sandwiched between two 50-ms masks consisting of random letters. No task was given to the masked distractor. After the first stimulus (experiment one) or distractor (experiment two), the screen was blank for 500 ms before the target was displayed for another 5000 ms or until response. The only difference between the two experiments was the masked distractor presentation. SOA: stimulus onset asynchrony

In each trial, participants responded to two consecutive Stroop stimuli, i.e., words whose colors were incongruent with their literal meaning, such as the word RED written in green (Fig. 1). Participants were asked to name the color when the word was underlined (color-naming) and to name the word when the word was not underlined (word-naming). Only word-color incongruent stimuli were used. When the two stimuli prompted different tasks (e.g., word-naming for the first, and color-naming for the second, or vice versa), the trial was considered task-switching. Otherwise, if the tasks were the same, it was a non-task-switching trial. Both the Stroop and the task-switching components were introduced to create task load differences.

Participants were instructed to respond as accurately as possible and were given ample time for each response (up to 5 s). Although participants needed to respond to both stimuli, only the second stimulus in a pair was considered the “target” for the purpose of our analysis because the task-switching effect and implicit interference only applied to this stimulus. Both reaction time and accuracy were measured for the target response. In the second experimental session, an additional gray word stimulus (17 ms) was added between the existing word stimuli with forward and backward masks (50 ms each). The masked word was either congruent or incongruent with the second word (target); congruent stimuli prompted the same response. However, no task was given to the masked word. Therefore, the explicit aspects of the tasks in each session were largely identical. More details of experimental design, rationale, and analysis are provided in the original study [2].

At the start of each session, participants underwent a short period of practice until they felt comfortable proceeding to the main experiment. The experiments consisted of three blocks of 64 trials. In the second session, a surprise post-experiment awareness test was performed to assess the invisibility of the masked words. During this test, participants were asked to judge the location (top/bottom) of the masked stimulus in a two-alternative forced-choice manner. Chance performance on this test indicated a lack of conscious awareness of the distractor.

### Electroencephalogram (EEG) recordings

The majority of participants had EEG data collected during the first experiment [5]. The data analysis is detailed in the original study, so here only critical information is provided. The signals were acquired with a 21-head-sensor dry electrode system [5–7]. Sensors were placed in five main locations, including the frontal, temporal, central, parietal, and occipital regions. The signals were sampled at 300 Hz and later bandpass filtered to exclude frequencies above 150 Hz and below 0.003 Hz. In the current study, we focused on alpha event-related desynchronization (ERD) because several of our previous studies showed that alpha ERD differentiated between CH-PATs and CH-NATs. Specifically, we previously identified stronger (more negative) alpha ERD in CH-PATs, as compared to CH-NATS, during working memory tasks [7] and Stroop tasks [5, 6], indicating hyperactivity during challenging tasks. We hypothesized that if alpha ERD reflects an attentional component during active engagement of a task, its value would correlate with implicit interference.

## Results

### Original key findings replicated: stronger implicit interference in CH-PATs

The analyses below are reported in the format of the mean (standard error of the mean [SEM]) unless otherwise specified. The implicit interference was deemed the target reaction time slowing caused by an incongruent implicit distractor, as compared to a congruent implicit prime. Because only thirty-six out of forty participants from the previous study were considered here, we repeated the same analyses to ensure that the results were similar. Indeed, the mean accuracy of the awareness test was 44.58% (2.32%) and slightly below chance (compared to 50%, *t*(29) = −2.33, *p* = 0.03), confirming the implicit nature of the distractor. No difference in distractor awareness was found between CH-NATs (44.26 (3.8) %) and CH-PATs (45.00 (2.21) %) (two-sample *t*-test, *t*(28) = 0.1543, *p* = 0.88). Similarly, we reproduced the key finding that during the high-load, color-naming condition, CH-PATs had more interference from an implicit response-incongruent distractor than CH-NATs (CH-PATs: 4.21 (1.51) % change in reaction time, *t*(18) = 2.79, *p* = 0.01; CH-NATs: −0.31 (1.1) % change in reaction time, *t*(16) = −0.28, *p* = 0.78). A direct comparison between the two yielded *t*(34) = 2.37, *p* = 0.02, Cohen’s *d* = 0.81.

### Practice has distinct effects on CH-PATs and CH-NATs

Both groups exhibited a practice effect in the second session. Overall, between the two sessions, the average reaction time decreased by 6.19% (3.04%), while the average accuracy increased by 6.09% (1.40%). The time interval between the two sessions was comparable across the two groups (CH-PATs: 15.84 (2.87) days; CH-NATs: 15.29 (1.68) days, *t*(34) = 0.16, *p* = 0.87).

We investigated whether practice over time differentially affects performance of the low-risk and high-risk participants. To this end, we first performed two separate mixed-effect analyses of variance (ANOVAs) on the *mean* accuracy and on the reaction time with one between-subject factor (participant status: CH-NATs/CH-PATs) and one within-subject factor (session number: one/two). The following data are collapsed across all three factors in the original design (task switching vs. non-switching, word-naming vs. color-naming, and incongruent vs. congruent distractors). Analysis of the accuracy yielded a main effect of session number, *F*(1, 34) = 21.66, *p* < 0.0001 , *η*_*p*_^*2*^ = 0.39, and an interaction between session and participants’ status, *F*(1, 34) = 9.03, *p* = 0.005 , *η*_*p*_^*2*^ = 0.21. The analysis of reaction time yielded a main effect of session, *F*(1, 34) = 4.20, *p* = 0.048 , *η*_*p*_^*2*^ = 0.11. Post hoc analyses revealed that CH-PATs exhibited a stronger practice effect in terms of accuracy (CH-NATs: 2.08% (1.36%) vs. CH-PATs: 9.67% (2.05 %), *t*(34) = −3.01, *p* = 0.005) but not reaction time (CH-NATs: 4.06% (4.44%) vs. CH-PATs: 8.10% (4.24%), *t*(34) = -0.66, *p* = 0.52).

**Fig. 2:**
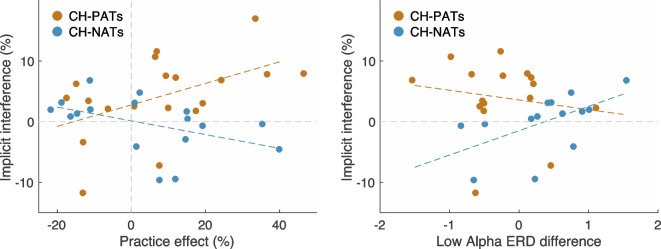
**Left** Distinct correlations between practice effect (percentage decrease of reaction time in the second session; positive values indicate a stronger practice effect, i.e., more decrease in reaction time, *x*-axis) and implicit interference (percentage decrease of reaction time between incongruent distractor and congruent prime; positive values indicate stronger interference, i.e., more increase in reaction time, y-axis) in CH-NATs (low-risk) and CH-PATs (high-risk). **Right** Positive correlation between lower alpha (8-11 Hz) ERD (event-related desynchronization) difference (*x*-axis) and the implicit interference (*y*-axis) was observed only in CH-NATs. Each dot represents a participant. The dotted lines were linearly fitted to the two correlations

To address the impact of practice on implicit interference, we ran a correlation analysis (Pearson’s correlation) between the implicit distractor interference effect and the practice effect in CH-NATs and CH-PATs, respectively. For the purpose of this analysis, the practice effect was defined as the percentage decrease of reaction time in the second session compared to the first because the interference effect was only observed in the domain of reaction time. Please note that the implicit distractor interference was calculated entirely based on the results of the second session. By converting reaction times into percentages for both interference and practice effects, baseline performance differences and the long tails in typical reaction time distributions were eliminated. Analysis of the CH-NATs yielded a marginal, negative correlation between practice and implicit interference (*r* = −0.46, *p* = 0.06). However, the same analysis on CH-PATs yielded a positive correlation between the two (*r* = 0.50, *p* = 0.03). We calculated *z*-scores for the *r* values and directly compared the two groups’ correlations with a two-tailed test, which resulted in a *p*-value of 0.004. These results showed that the interaction between practice and the implicit interference was distinct between CH-NATs and CH-PATs: better performance in the second session was correlated with less implicit distraction in CH-NATs, while for CH-PATs, better performance in the second session led to stronger distraction (Fig. 2, left).

### Greater low-range alpha ERD difference between task-switching and non-switching trials on EEG in session one is correlated with interference in CH-NATs

**Fig. 3:**
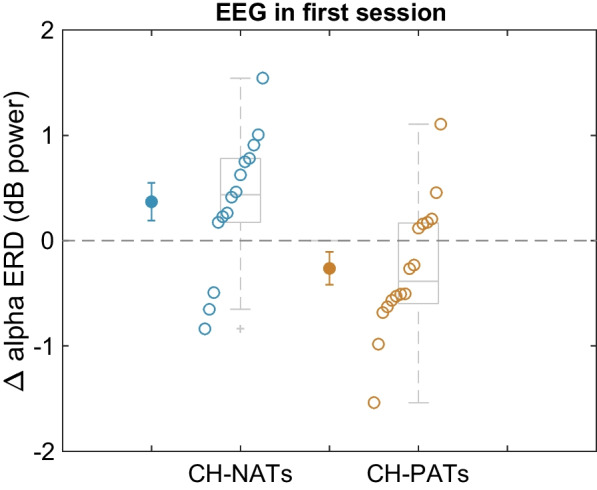
Alpha ERD difference between task-switching and non-switching trials in session one in CH-NATs and CH-PATs. On average, CH-PATs exhibited a stronger ERD (reduction in power) during task-switching than non-switching trials, while CH-NATs showed the opposite

In the first session, EEG data was analyzed for the majority of participants (14 of 17 CH-NATS and 16 of 19 CH-PATs), which allowed us to determine the correlation between prior EEG data and later behavioral performance in these two groups. To minimize multiple comparisons and to focus on an attention component, we examined the most distinctive alpha event-related desynchronization (ERD) difference between the CH-NATs and CH-PATs. In the original study, we calculated the difference in the central area (C3, Cz, C4) alpha ERD between high-load task-switching trials and low-load non-switching trials (alpha ERD (switching)–alpha ERD (non-switching)). Note that ERD is a reduction of power, and a stronger (more negative) ERD is therefore a stronger reduction. For CH-PATs, the alpha ERD difference was negative, indicating that these participants had stronger low alpha ERDs during switching trials than non-switching trials. For CH-NATs, the opposite was observed: their alpha ERD difference was positive, showing stronger low alpha ERDs during non-switching trials. Together, these results suggest that CH-PATs have stronger attentional engagement during task switching than on non-switching tasks, whereas CH-NATs have more engagement when not changing tasks. These results were also found with the reduced cohort in our study (low alpha ERD mean difference (*SD*), CH-PATs: −0.26 (0.63) vs. CH-NATs: 0.37 (0.67), *t*(28) = −2.68, *p* = 0.01, Cohen’s *d* = 1.00) (Fig. 3).

We ran a correlation analysis between the aforementioned low alpha ERD difference in the first session and the implicit interference in the second session in both groups. CH-PATs had no correlation between their low alpha ERD difference and implicit interference (*r* = −0.16, *p* = 0.54). In contrast, there was a positive correlation between low alpha ERD difference and implicit interference in CH-NATs (*r* = 0.56, *p* = 0.04) (Fig. 2, right). A further *z*-score comparison between the two correlations revealed a *p*-value of 0.05. These results showed that the CH-NATs who exhibited a higher low-range alpha ERD difference in the first session also experienced stronger distraction in the second session. This finding suggests that in low-risk participants, having lower attentional engagement in switching trials was associated with stronger implicit interference. A framework for how CH-PATs and CH-NATs utilized attentional resources differently is provided in "Discussion" section.

## Discussion

In our two original studies, we examined the electrophysiological signatures [5] during a cognitively demanding task as well as the behavioral response to implicit distracting information [2] in cognitively healthy older individuals with a high risk (CH-PATs) or low risk (CH-NATs) of cognitive decline. The largely identical experimental designs and participants of these studies created a unique opportunity for the current study to investigate if practice or the EEG signatures found in the first session were associated with implicit interference. Overall, we found that practice had distinct effects on high-risk and low-risk individuals. While the practice effect was negatively correlated with interference in the low-risk participants, a positive correlation was found in the high-risk participants. Furthermore, in low-risk participants, stronger interference was associated with a greater low-range alpha ERD difference between task-switching and non-switching trials, suggesting that lower attentional engagement in switching trials is associated with susceptibility to interference. Although the high-risk participants exhibited similar implicit inteference to what has been observed in a healthy young population [4], our study provides evidence that an increase in implicit interference with decreased task load is not a part of normal aging. Instead, these changes provide a potential indicator for risk of cognitive decline.

Our original study showed that, albeit being cognitively healthy, high-risk individuals were distracted to a higher extent by a subliminal cue [2]. One likely explanation of this phenomenon is that directing additional attention toward the sensory environment could help maintain task performance and keep high-risk individuals overtly cognitively healthy, but this strategy comes at the expense of processing irrelevant sensory information when subliminal stimuli are incongruent. This interpretation suggests an active processing of subliminal information in high-risk individuals, which is further supported in the current study. The opposite correlations between practice and implicit interference in low-risk and high-risk individuals indicate distinct underlying cognitive infrastructures in these two populations. If practice reduces cognitive load, individuals should have more cognitive resources available in the second session. Based on our results, low-risk and high-risk individuals utilized these additional cognitive resources differently. Low-risk individuals learned to better inhibit distracting implicit information, while more practice in high-risk individuals led to stronger interference, possibly due to additional processing of distracting implicit information. The higher attentional commitment in high-risk individuals is demonstrated by a stronger alpha ERD compared to low-risk individuals during various challenging tasks [5–7], including the first session of our study. In the second session, we speculate that high-risk individuals used the cognitive resources that were freed by practice to take in additional sensory information such as subliminal cues. Future research is needed to unravel the neural machinery of the attention network in high-risk individuals.

We believe that the opposite effects of practice on implicit interference in high-risk and low-risk populations provide valuable data for working toward possible future interventions. Theoretically, practice leads to an increase in available attentional resources due to decreased task load. The key finding of the current study is that the use of this additional attention differs in high- and low-risk populations, which could signal different cognitive or perceptual strategies. Recently, a number of studies have conducted cognitive training as well as repetitive transcranial magnetic stimulus (rTMS) as interventions to combat the cognitive decline in Alzheimer’s disease [9–11]. Specifically, most of these studies focused on improving higher-level cognitive abilities, such as memory or language, that are directly relevant to the clinically observable cognitive decline in Alzheimer’s. However, our findings suggest that cognitive decline at earlier stages could be perceptual; that is, the decline could influence how one’s sensory system selects and suppresses information in the environment. Therefore, can we minimize the effects of this decline by training perceptual pathways in high-risk individuals? Visual perceptual learning, defined as long-term improvement resulting from visual experience, has shed light on this question. In the domain of visual perceptual learning, behavioral and neural improvement have been shown toward imperceptible visual stimuli after a prolonged training period. Chang et al. [12] showed post-training improvement in a coherent motion discrimination task in both younger and older participants. Importantly, both groups showed improvement when the motion was sub-threshold (i.e., not consciously perceived). Learning plasticity and flexibility of visual processing in the older individuals have also been demonstrated through structural changes in the underlying visual pathway. Yotsumoto et al. [13] showed that after a 3-day texture discrimination training, both older and younger participants exhibited behavioral improvement. However, only older participants exhibited white matter fiber density changes in the early visual areas (i.e., V1-V3) as measured by fractional anisotropy. Visual perceptual learning thus provides a promising approach for training the older individuals to use specific information, whether supraliminal or subliminal, to support their performance. Critically, the finding that older individuals can learn from irrelevant information that younger individuals suppress [12] sheds light on the importance of inhibitory control in filtering and encoding external information in the older individuals.

In low-risk individuals, the positive correlation between low alpha ERD difference between task-switching and non-switching trials in the first session and implicit interference in the second session illustrates the similarities between low-risk cognitively healthy older adults and healthy young participants. That is, when low-risk participants are more attentive to a target, implicit interference is less likely to occur, just as in younger individuals. Recently, with concurrent tracking of target- and distractor-triggered alpha oscillations, Gutteling and colleagues [14] showed that distractor-triggered alpha oscillations increased significantly when the target was more difficult to process. These results from young individuals offer another explanation regarding distractors: task load could modulate alpha power in response to both distractors and targets. Future research on older individuals is needed to disentangle the target and distractor effects and to strengthen the current results with a larger sample size.

Taken together, however, our results provide evidence that practice, although typically considered a positive effect, may uniquely influence high-risk individuals by increasing susceptibility to implicit interference. In low-risk participants, however, practice appears to remain positive as their behavioral and neurophysiological data demonstrate high similarity with that of younger individuals. How and when the practice effects of the two groups bifurcate is a critical research question for better understanding early cognitive decline in Alzheimer’s.

### Supplementary information

ESM 1 (DOCX 38.2 kb)

## Data Availability

The current study was not preregistered. The raw data and code are available upon reasonable request.
