# Supplementary material for: Practice makes imperfect: stronger implicit interference with practice in individuals at high risk of developing Alzheimer’s disease
Source: GeroScience. 2023 Oct 10;46(2):2777–86. doi: 10.1007/s11357-023-00953-9 (PMC10828369; doi:10.1007/s11357-023-00953-9)
Supplement: Supplementary file 1 — (DOCX 38.2 kb) [file 11357_2023_953_MOESM1_ESM.docx]

**Supplementary material**

**Practice makes imperfect: stronger implicit interference with practice in individuals at high risk of developing Alzheimer’s disease**

Shao-Min Hung^1,2,3*ψ^, Sara W. Adams^3ψ^, Cathleen Molloy^4^, Daw-An Wu^3^, Shinsuke Shimojo^3,5^, Xianghong Arakaki^4^

^1^Waseda Institute for Advanced Study, Waseda University, Tokyo, Japan

^2^Faculty of Science and Engineering, Waseda University, Tokyo, Japan

^3^Biology and Biological Engineering, California Institute of Technology, Pasadena, CA, USA

^4^Cognition and Brain Integration Laboratory, Department of Neurosciences, Huntington Medical Research Institutes, Pasadena, CA, USA

^5^Computation and Neural Systems, California Institute of Technology, Pasadena, CA, USA

^ψ^These authors contributed equally.

*Correspondence: [smhung@aoni.waseda.jp](mailto:smhung@aoni.waseda.jp)

Content

1. Examination of individual awareness test performance.

2. Examination of Stroop and task-switching effects in the current dataset.

3. Complete ANOVA results of the practice effect in ACC and RT

4. Classification of CH-PATs and CH-NATs**1.** Examination of individual awareness test performance.

At the group level, the mean accuracy of the awareness test was 44.58% (2.32%) and slightly below chance (compared to 50%, *t*(29) = -2.33, *p* = 0.03), corroborating the implicit nature of the distractor. No difference in distractor awareness was found between CH-NATs (44.26 (3.8) %) and CH-PATs (45.00 (2.21) %) (two-sample t-test, *t*(28) = 0.1543, *p* = 0.88). To examine the possibility of a subgroup of individuals with higher than chance performance skewing the data, we ran analyses that excluded individuals who had an accuracy above 60%. This left us with 16 CH-PATs and 16 CH-NATs (Figure 1S). We first replicated our main finding in the previous study; that is, CH-PATs, but not CH-NATs, slowed down with an implicit distractor in the high-load task (color-naming: CH-PATs: 4.00% RT increase; CH-NATs: 0.76 % RT decrease. *T*(34) = 2.28, *p* = 0.03.) but not low-load task (word-naming: CH-PATs: 1.98% RT decrease; CH-NATs: 2.60 % RT increase. *T*(34) = -1.87, *p* = 0.07.) The correlation between practice and the implicit effect was smaller, as can be expected with a smaller sample size. Analysis of the CH-NATs yielded no correlation between practice and implicit interference (*r* = -0.32, *p* = 0.23). However, the same analysis on CH-PATs yielded a marginally positive correlation between the two (*r* = 0.45, *p* = 0.08), which aligns with the results found without excluding participants. We calculated z-scores for the r values and directly compared the two groups’ correlations with a two-tailed test, which resulted in a *p*-value of 0.04.

Fig. 1S. Individual mean accuracy on post-experiment awareness test (n = 16 CH-PATs and 16 CH-NATs). 50% indicates chance performance. Trials with no response were excluded from this analysis. Some participants refused to complete the task, leading to no or extremely low accuracy values (3 CH-PATs and 4 CH-NATs); however, their data was still included in the present study to preserve the sample sizes.

2. Examination of Stroop and task-switching effects in the current dataset.

In the main text, we reported successful replications of the main findings from the original study. Here we further examined the Stroop, task switching, implicit interference, and individual risk status in a complete mixed-effect analysis of variance based on reaction time. The analysis yielded main effects of task switching, *F*(1, 34) = 46.93, *p* = .000, *η*p2 = 0.58; Stroop, *F*(1, 34) = 5.79, *p* = .02, *η*p2 = 0.15; distractor congruency, *F*(1, 34) = 3.41, *p* = .07 (marginal), *η*p2 = 0.09, and no effect of participant status, *F*(1, 34) = 0.15, *p* = .70, *η*p2 = 0.00. Intriguingly, a three-way interaction was observed across participant status, task-switching, and distractor incongruency, *F*(1, 34) = 4.51, *p* = .04, *η*p2 = 0.09. These results largely replicated our original findings in a smaller participant cohort and suggested that task switching could be a factor for attentional modulation.

3. Complete ANOVA results of the practice effect in ACC and RT

| **ACC** | **SumSq** | **DF** | **MeanSq** | **F** | ***p* value** |
| --- | --- | --- | --- | --- | --- |
| Intercept | 57.686 | 1 | 57.686 | 4884.3 | 2.653e-38 |
| Risk level | 0.012 | 1 | 0.012 | 0.98 | 0.33 |
| Error | 0.402 | 34 | 0.012 |  |  |
| Session (intercept) | 0.062 | 1 | 0.062 | 21.66 | 4.7981e-05 |
| Risk level x session | 0.026 | 1 | 0.026 | 9.03 | 0.005 |
| Error (session) | 0.097 | 34 | 0.003 |  |  |

| **RT** | **SumSq** | **DF** | **MeanSq** | **F** | ***p* value** |
| --- | --- | --- | --- | --- | --- |
| Intercept | 134.4 | 1 | 134.4 | 690.59 | 3.6006e-24 |
| Risk level | 0.080 | 1 | 0.080 | 0.41 | 0.525 |
| Error | 6.617 | 34 | 0.195 |  |  |
| Session (intercept) | 0.157 | 1 | 0.157 | 4.20 | 0.048 |
| Risk level x session | 0.016 | 1 | 0.016 | 0.43 | 0.520 |
| Error (session) | 1.271 | 34 | 0.037 |  |  |

4. Classification of CH-PATs and CH-NATs

The CSF A*β*_42_ / total tau ratio cutoff that was used to differentiate CH-PATs and CH-NATs originated from our earlier study identifying differences between Alzheimer’s patients and non-Alzheimer’s patients (AD vs non-AD) [1]. The analyses are detailed in the original study, which included 29 individuals with dementia, 40 with mild cognitive impairment, and 70 who were cognitively healthy. We first derived an A*β*_42_ / total tau ratio cutoff that had 85% sensitivity and 64% specificity for classifying individuals into AD or non-AD groups. Subsequently, CH-PATs and CH-NATs were identified from the 70 cognitively healthy individuals using the same cutoff. That is, CH-PATs had similar CSF A*β*_42_ and total tau concentrations to the AD group, and thus were defined as preclinical AD/high-risk.

Whether the classification of CH-PATs and CH-NATs is indeed correct does require longitudinal follow-up of these individuals. Our preliminary four-year longitudinal data [2] with 34 CH-PATs and 34 CH-NATs at the first time point and 28 CH-PATs and 32 CH-NATs at the second time point showed promising results. None of the CH-NATs had cognitive decline, while 11 of 28 CH-PATs developed cognitive impairment.

References

1 Harrington MG, Chiang J, Pogoda JM, Gomez M, Thomas K, Marion SD, et al. Executive Function Changes before Memory in Preclinical Alzheimer’s Pathology: A Prospective, Cross-Sectional, Case Control Study. PLoS ONE. 2013 Nov;8(11):e79378.

2 Harrington MG, Edminster SP, Buennagel DP, Chiang JP, Sweeney MD, Chui HC, et al. Four-year longitudinal study of cognitively healthy individuals: csf amyloid/tau levels and nanoparticle membranes identify high risk for Alzheimer’s disease. Alzheimer’s & Dementia: The Journal of the Alzheimer’s Association. 2019 Jul;15(7):P299.
